# Gender differences in the mediating effect of physical activity on the relationship between self-efficacy and subjective exercise experiences among Chinese college students: based on social cognitive theory

**DOI:** 10.3389/fpsyg.2026.1759512

**Published:** 2026-01-20

**Authors:** Ying Zhao, Qinghua Wu, Pan He

**Affiliations:** 1 School of Physical Education and Health, Sanming University, Sanming, China; 2 School of Basic Education, Guangdong Finance & Trade Vocational College, Qingyuan, China; 3 College of Humanities, Yiyang Medical College, Yiyang, China

**Keywords:** college student, gender difference, physical activity, self-efficacy, subjective exercise experience

## Abstract

Chinese undergraduates exhibit notable gender differences in their subjective exercise experiences. These differences are closely linked to variations in their physical activity participation and self-efficacy beliefs. Despite growing research on the links between self-efficacy, physical activity, and subjective exercise experiences, few studies have systematically examined whether physical activity mediates the relationship between self-efficacy and subjective exercise experiences. Guided by social cognitive theory (SCT), this study aims to investigate the mediating effect of physical activity on the relationship between self-efficacy and subjective exercise experiences and further explore potential gender differences in this mediating mechanism among Chinese college students. This study conducted an online questionnaire survey among 1,674 college students (*n* = 1,674; M = 19.15, SD = 1.23; 506 males and 1,186 females). Structural equation modeling (SEM) revealed physical activity partially mediated the links between self-efficacy and positive well-being (*β* = 0.10, *p* < 0.01), and self-efficacy and fatigue (*β* = 0.13, *p* < 0.01), but not self-efficacy and psychological distress (*β* = 0.04, *p* = 0.22). This study confirms physical activity partially mediates the link between self-efficacy and Chinese college students’ subjective exercise experiences (positive well-being, fatigue), with notable gender differences. These findings support gender-specific exercise interventions: enhancing self-efficacy to increase physical activity for males, and focusing on self-efficacy to reduce negative experiences for females.

## Introduction

Physical activity is important for the health and well-being of college students but many students around the world do not get enough physical activity (Jago et al., 2023; White et al., 2017). Studies show that a large number of college students fail to meet global recommendations for physical activity (Guthold et al., 2018). This lack of activity means students miss out on the positive psychological benefits of exercise, which can also make it harder for them to build lasting exercise habits (Li et al., 2018). How students feel when they exercise, known as their subjective exercise experience, is very important. These feelings can influence whether they want to continue being active. Understanding why these gender differences exist is a key step toward helping all students.

Social cognitive theory (SCT) helps us understand these differences and this theory suggests that a person’s belief in their own ability, called self-efficacy, is a powerful driver of their health behaviors and the outcomes of those behaviors (Bandura, 1997). Self-efficacy can influence how much physical activity a person does, which can also directly influence how they feel during that activity. This study aims to fill these gaps Guided by SCT by investigating whether physical activity acts as a mediator between self-efficacy and three exercise experiences: positive well-being, psychological distress, and fatigue. In addition, we will explore if there are significant gender differences in this entire process.

The results of this study will provide new insights for tailored programs to encourage physical activity. By understanding the unique pathways for male and female students, universities can design specific interventions. Ultimately, this research can help Chinese college students become more active and enjoy the many benefits of physical activity.

## Literature review

### The prevalence and consequences of inadequate physical activity among college students

Inadequate physical activity is a widespread and pressing public health issue among college students globally (Guthold et al., 2018) that many students do not meet the recommended levels of physical activity. The WHO advises that adults aged 18 to 64 should engage in at least 150 min of moderate-intensity or 75 min of vigorous-intensity physical activity per week (van Sluijs et al., 2021). However, a significant number of college students fall short of this guideline. Global studies indicate that a large proportion of this population is insufficiently active (Chaput et al., 2020; Guthold et al., 2018). This trend is also evident in China, where research shows that Chinese college students often exhibit low levels of physical activity, with academic pressures and increased screen time being significant contributing factors (Yan et al., 2024; Zhu et al., 2017).

The consequences of this physical inactivity are serious and affect both physical and mental health (Hong et al., 2020). On a physical level, low physical activity is a well-known risk factor for the development of non-communicable diseases (van Sluijs et al., 2021). For students, it can lead to reduced physical fitness, lower energy levels, and poorer overall health. The transition to university life often involves changes in lifestyle that can exacerbate these risks.

Perhaps more immediately relevant to the daily lives of students are the psychological consequences. Inadequate physical activity is strongly linked to poorer mental health outcomes, such as higher risk of developing symptoms of depression and anxiety (Kandola et al., 2019). Furthermore, when students are not active enough, they miss out on the well-established psychological benefits of exercise. Regular physical activity is known to improve mood, reduce stress, and enhance subjective well-being (Eather et al., 2023; Warburton and Bredin, 2017). Therefore, a lack of activity can mean fewer positive emotional experiences and a reduced capacity to cope with the demands of university life.

This lack of positive reinforcement from physical activity can create a negative cycle. If a student does not feel the psychological benefits, their motivation to be active may decrease (Ryan and Deci, 2000). This makes it harder for them to adopt and maintain a consistent exercise routine over the long term. This cycle helps explain why physical inactivity can become a persistent problem during the college years and beyond (Dishman et al., 2006).

Thus, the high prevalence of inadequate physical activity among college students are a major concern. It leads to significant negative consequences for their physical health and mental well-being and also prevents them from gaining the positive experiences that would help them build lasting healthy habits.

### Social cognitive theory and its application to physical activity behavior

Social cognitive theory (SCT) is the well-established framework for understanding human behavior (Bandura, 1997). This theory suggests that personal factors, environmental influences, and behavior itself all interact in a dynamic way. This is known as triadic reciprocal determinism and the central concept within SCT is self-efficacy.

Self-efficacy refers to a person’s belief in their own ability to plan and execute the courses of action required to achieve specific goals (Bandura, 1997). It is not about a person’s actual skills, but rather their confidence in using those skills in particular situations (Bandura and Wessels, 1994). In the context of physical activity, self-efficacy would be an individual’s confidence in their ability to be physically active, even when facing challenges like a lack of time, bad weather, or feeling tired (McAuley and Blissmer, 2000; Warner et al., 2014).

According to SCT, self-efficacy influences behavior in several important ways such as affecting the goals, effort, and persist (Ashford et al., 2010). A person with high self-efficacy for exercise is more likely to set challenging activity goals, put in strong effort, and not give up easily. On the other hand, a person with low self-efficacy may avoid physical activity altogether or stop quickly if they find it difficult (Bandura, 2004).

Research strongly supports the role of self-efficacy in promoting physical activity. For example, a review study found that higher levels of self-efficacy were consistently linked to greater participation in physical activity across different age groups (Olander et al., 2013). Another study focusing on college students showed that self-efficacy was a key factor in predicting whether students started and maintained an exercise program (Rovniak et al., 2002). This is because students who believe they can overcome barriers are more likely to translate their intentions into actual behavior.

SCT offers a powerful lens for understanding physical activity behavior, which positions self-efficacy as a core driver, influencing whether individuals choose to be active. This theoretical foundation is essential for designing effective interventions aimed at increasing physical activity levels.

### Physical activity and positive exercise experiences

Engaging in adequate physical activity is closely linked to a range of positive subjective experiences during and after exercise. These experiences are crucial for understanding why some people maintain an active lifestyle while others do not. Positive exercise experiences generally include feelings of positive well-being, such as energy, joy, and revitalization (Reed and Ones, 2006). They also involve a reduction in negative states like psychological distress and fatigue (Gauvin and Spence, 1998).

A substantial body of research supports the immediate psychological benefits of single sessions of physical activity. This is often called the “feel-good effect.” Even a single bout of moderate intensity exercise can lead to noticeable improvements in mood and a decrease in feelings of stress and tension (Basso and Suzuki, 2016). For example, studies have shown that people report feeling more enthusiastic, alert, and less anxious after a workout. This immediate positive feedback makes the exercise experience itself more rewarding and can strengthen the desire to repeat the activity (Reed and Ones, 2006).

Engaging in adequate physical activity is closely linked to a range of positive subjective experiences during and after exercise. From the perspective of SCT, these positive experiences are not merely automatic outcomes of movement. Instead, they are significantly influenced by an individual’s cognitive processes, particularly their self-efficacy beliefs (Bandura, 1997). When people with high self-efficacy engage in physical activity, they are more likely to interpret physical sensations, such as an increased heart rate and sweating, as signs of accomplishment and improved fitness rather than as unpleasant strain. This positive cognitive appraisal during exercise is a key mechanism that directly fosters feelings of well-being and reduces distress (McAuley and Blissmer, 2000).

Physical activity is a powerful driver of positive subjective exercise experiences (He et al., 2022). Through the lens of SCT, these benefits (e.g., enhanced well-being, lower psychological distress, and reduced fatigue) are strongly shaped and amplified by an individual’s self-efficacy (Buecker et al., 2021). These positive experiences are critical because they serve as reinforcing outcomes, helping to build the motivation needed to sustain a physically active lifestyle over the long term (Williams et al., 2018).

### Gender differences in physical activity and exercise experiences

Consistent evidence shows that male and female college students often differ in their physical activity behaviors and their subjective experiences during exercise. Understanding these gender differences is crucial for developing effective health promotion strategies.

Prior studies have repeatedly found that male students generally report higher levels of physical activity than female students (Guthold et al., 2018; Liu et al., 2023). Males are more likely to participate in moderate to vigorous activities and to engage in sports. In contrast, female students often show a decline in physical activity during the transition to young adulthood. This gap in participation is a key starting point for understanding subsequent differences in exercise-related experiences.

These behavioral differences are closely linked to disparities in self-efficacy, which posits that self-efficacy is a primary determinant of behavior. Studies confirm that male adolescents and young adults often report higher self-efficacy for physical activity than their female counterparts (Li et al., 2018). This means that male students express greater confidence in their ability to participate in sports, overcome barriers like lack of time, and persist with exercise even when it is difficult. This stronger sense of efficacy is a powerful driver of their more active lifestyles.

As a result of these differences in behavior and self-efficacy, the subjective exercise experiences of male and female students also tend to diverge. Male students frequently report higher levels of positive well-being, such as energy and enjoyment, from physical activity. Female students, however, often report experiencing more psychological distress and higher levels of fatigue during and after exercise (Biedenweg et al., 2014). This pattern suggests that the same activity can be perceived and processed differently by men and women.

In conclusion, significant gender differences exist in physical activity, self-efficacy, and subjective exercise experiences among college students. These differences are not merely biological but are powerfully shaped by social, cognitive, and environmental factors as explained by SCT. This underscores the importance of examining the psychological pathways between self-efficacy, behavior, and experience separately for men and women, which is a central aim of the present study.

### The current research

As the literature review has established, self-efficacy is a key predictor of physical activity, which in turn is linked to subjective exercise experiences. Furthermore, notable gender differences exist across these variables. However, a significant gap remains in our understanding that physical activity serves as the mediating mechanism between self-efficacy and different exercise experiences. More importantly, it is not known whether this entire process functions differently for male and female students.

**Figure 1:**
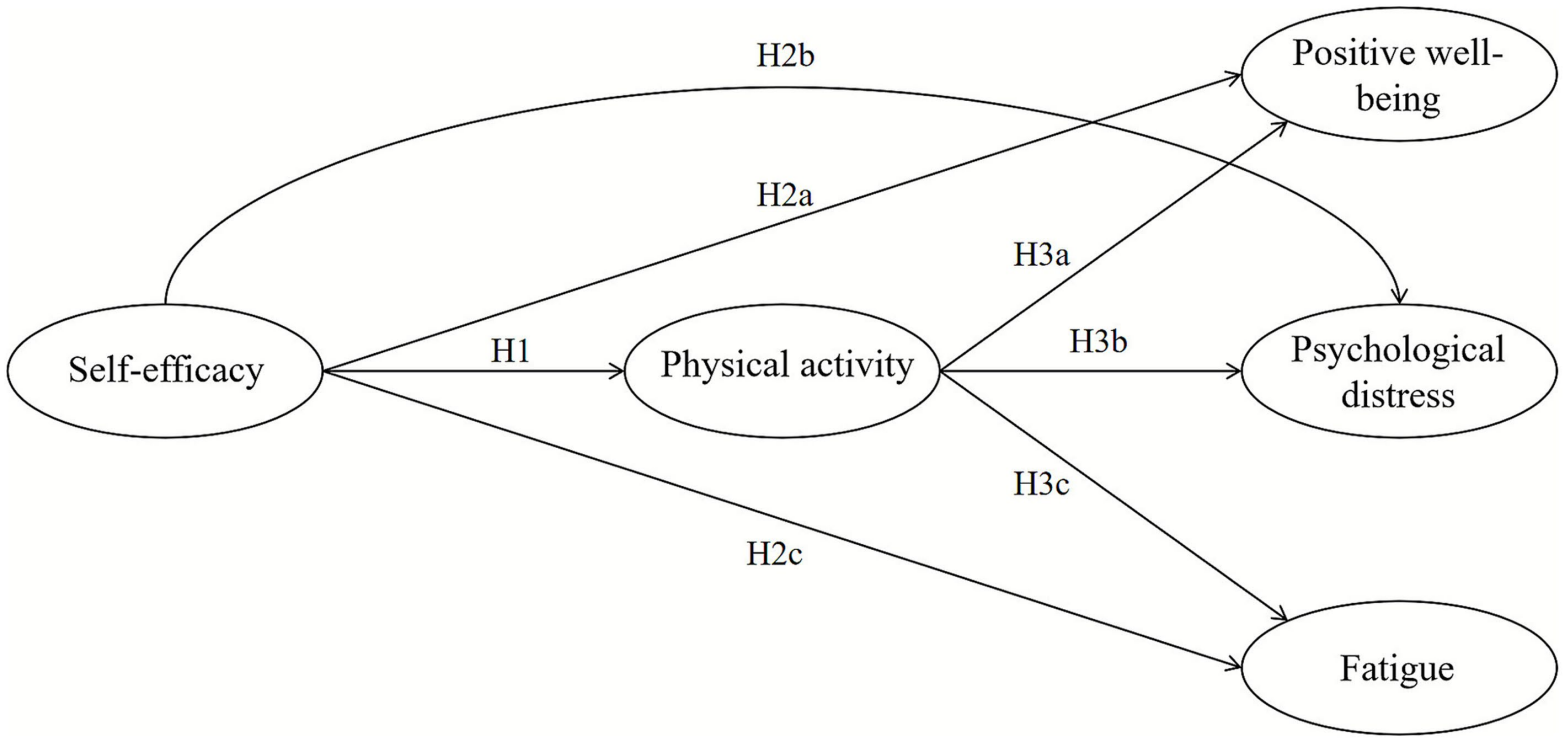
Hypothesis model.

Guided by SCT, we will construct and test a model in which physical activity mediates the relationship between self-efficacy and three dimensions of subjective exercise experience: positive well-being, psychological distress, and fatigue. A key strength and novelty of our study is the use of multi-group structural equation modeling to explicitly test for gender differences in this mediating mechanism. Based on the theoretical framework and existing empirical evidence, we propose the following hypotheses, which are also presented in Figure 1.

This study examines (as shown in Figure 1):

*H1*: Self-efficacy positively influences undergraduates’ physical activity.

*H2*: Self-efficacy influences undergraduates’ subjective exercise experience.

*H2a*: Self-efficacy positively influences undergraduates’ positive well-being.

*H2b*: Self-efficacy positively influences undergraduates’ psychological distress.

*H2c*: Self-efficacy negatively influences undergraduates’ fatigue.

*H3*: Physical activity influences undergraduates’ subjective exercise experience.

*H3a*: Physical activity positively influences undergraduates’ positive well-being.

*H3b*: Physical activity negatively influences undergraduates’ psychological distress.

*H3c*: Physical activity positively influences undergraduates’ fatigue.

## Methods

### Participants

This research recruited a total of 2,216 participants using convenience sampling methodology. The final sample is 1,674 (M = 19.15, SD = 1.23; 506 males and 1,186 females) after excluding the blank questionnaire or the questionnaire with same response. The sample size of this study satisfies the minimum requirement for structural equation modeling as suggested by prior research (Xiong et al., 2015).

### Measures

#### Self-efficacy

This study employed the Chinese version of the *Self-efficacy* (Chen et al., 2019), which contains 8 item, such as “I have the coordination I need to be physically active during my free time on most days.” Participants rated each item on a 5-point Likert scale ranging from 1 (“Disagree a lot”) to 5 (“Agree a lot”). The reliability (*α* = 0.89) and CFA results indicate structural validity of the modified scale are acceptable (*χ*^2^ = 203.74, RMSEA = 0.09, CFI = 0.94, TLI = 0.92, SRMR = 0.04).

#### Subjective Exercise Experiences Scale

To assess participants’ global psychological responses to exercise, this study adopted the *Subjective Exercise Experiences Scale (SEES)* (MeAuley and Courneya, 1994). The SEES consists of 12 items grouped into three distinct dimensions, each with 4 items: positive well-being (PWB, α = 0.81), psychological distress (PD, *α* = 0.88) and fatigue (*α* = 0.90). Participants rated each item on a 7-point Likert scale, where 1 = “Not at all” and 7 = “Very much so.” CFA was conducted to verify the factorial validity of the scale in the current sample, yielding acceptable model fit [*χ*^2^ (76) = 454.00, RMSEA = 0.05, CFI = 0.95, TLI = 0.94, SRMR = 0.07].

#### Physical activity

Physical activity was assessed using the International Physical Activity Questionnaire-Short Form (IPAQ-SF) (Craig et al., 2003), a widely validated and internationally recommended instrument for quantifying habitual physical activity and sedentary behavior in adult populations. The IPAQ-SF comprises 7 items: 6 items capture participants’ engagement in different types of physical activity (i.e., vigorous-intensity, moderate-intensity, and walking), while 1 item assesses the duration of sedentary behavior (e.g., sitting or reclining) on a typical day. Consistent with standard IPAQ scoring protocols and previous empirical applications (Esmaeilzadeh et al., 2022), weekly physical activity volume was quantified using metabolic equivalent of task (MET)-minutes, calculated as the sum of weighted values for each activity type: 8.8 METs (vigorous-intensity activity) × weekly frequency (days/week) × daily duration (minutes/day) + 4.0 METs (moderate-intensity activity) × weekly frequency (days/week) × daily duration (minutes/day) + 3.3 METs (walking, classified as moderate-intensity) × weekly frequency (days/week) × daily duration (minutes/day). In the current study, to ensure data quality and adherence to statistical assumptions, MET-minute values were standardized prior to subsequent analyses. Extreme outliers (values exceeding ±3.29 standard deviations) were identified and excluded using established criteria (Osborne, 2012), as such values may introduce bias in parametric statistical models.

#### Demographic variables

Demographic variables included participants’ school affiliation, gender, age, height, and weight.

### Procedure

This study was approved by the Institutional Review Board (IRB) of Sanming University (Approval No. 2025ACNN-006) prior to data collection. Data were gathered from undergraduates at a university located in South China in September 2025. A total of 2,216 college students were recruited via *Wenjuanxin*, an online questionnaire platform widely used in academic research in China.

Given that all participants were adults, written informed consent was obtained directly from each participant prior to their participation in the survey. Each participant completes the survey independently within 10–15 min. To enhance response rate and completion quality, participants received a small incentive (e.g., stationery, a coffee voucher) upon successful submission of valid questionnaires.

All data required to replicate the study’s findings have been deposited in the ScienceDB repository (DOI: https://doi.org/10.57760/sciencedb.31863) and are publicly accessible for non-commercial academic research purposes only, in adherence to open science practices and reproducibility standards in empirical research.

### Statistical analysis

SPSS 25.0 was employed for preliminary data screening and descriptive analyses, including the computation of means, standard deviations, and frequency distributions for all study variables. This software was further utilized to conduct Pearson correlation analyses, internal consistency reliability assessments via Cronbach’s *α* coefficients, and common method bias (CMB) detection. Specifically, Harman’s single-factor test was performed to identify potential CMB: all scale items were subjected to unrotated exploratory factor analysis, and a single factor accounting for > 40% of the total variance was considered indicative of substantial bias (Podsakoff et al., 2003).

Mplus 8.3 was used for advanced psychometric and structural model analyses. First, confirmatory factor analysis (CFA) was conducted to assess the factorial validity of all measurement scales. Model fit was evaluated using a suite of complementary indices to mitigate the limitations of sole reliance on the chi-square (*χ*^2^), comparative fit index (CFI), Tucker–Lewis index (TLI), root mean square error of approximation (RMSEA), and standardized root mean square residual (SRMR). Consistent with widely accepted benchmarks (Hu and Bentler, 1999), good model fit was defined as CFI ≥0.95, TLI ≥0.95, RMSEA ≤0.06, and SRMR ≤0.08. Subsequent hypothesis testing of the structural model and measurement invariance were also executed in Mplus 8.3. Missing data were addressed using the software’s default full information maximum likelihood (FIML) estimation method.

## Results

### Common methods bias

Common method bias (CMB) refers to the systematic error variance introduced when data for all study variables are collected through a single measurement method, such as self-report questionnaires, which may distort the relationships between constructs. This study assessed potential CMB using Harman’s single-factor test, a widely employed statistical approach for detecting such bias (Podsakoff et al., 2003). All scale items were subjected to an unrotated exploratory factor analysis with principal component extraction and the results showed that the percentage of total variance explained by the first factor was 30.52%. This value is below the critical threshold of 40%, indicating that CMB was not a serious concern in this study.

### Descriptive statistics

As shown in Table 1, Pearson correlation analyses revealed several significant relationships. Self-efficacy was positively correlated with positive well-being (*r* = 0.44, *p* < 0.01) and physical activity (*r* = 0.42, *p* < 0.01), and negatively correlated with psychological distress (*r* = −0.33, *p* < 0.01) and fatigue (*r* = −0.31, *p* < 0.01). Positive well-being was negatively correlated with psychological distress (*r* = −0.49, *p* < 0.01) and fatigue (*r* = −0.32, *p* < 0.01). Psychological distress and fatigue showed a strong positive correlation (*r* = 0.62, *p* < 0.01). Gender was significantly correlated with all main variables. Specifically, being female was associated with lower self-efficacy (*r* = −0.30, *p* < 0.01), lower positive well-being (*r* = −0.15, *p* < 0.01), higher psychological distress (*r* = 0.19, *p* < 0.01), higher fatigue (*r* = 0.12, *p* < 0.01), and lower physical activity (*r* = −0.33, *p* < 0.001). Age showed weak but significant correlations with several variables, including positive well-being (*r* = 0.07, *p* < 0.01), psychological distress (*r* = −0.12, *p* < 0.01), fatigue (*r* = −0.15, *p* < 0.01), and physical activity (*r* = 0.09, *p* < 0.01).

**Table 1:** Results of descriptive statistics.

	Variables	M	SD	Ske.	Kur.	*α*	1	2	3	4	5	6
1	Self-efficacy	3.03	0.74	−0.01	0.07	0.89						
2	Positive well-being	4.48	1.09	0.05	0.55	0.81	0.44^***^					
3	Psychological distress	2.94	1.19	0.40	0.09	0.88	−0.33^***^	−0.49^***^				
4	Fatigue	4.23	1.37	−0.19	−0.11	0.90	−0.31^***^	−0.32^***^	0.62^***^			
5	Physical activity	−0.08	0.71	0.06	3.47	/	0.42^***^	0.28^***^	−0.16^***^	−0.07^**^		
6	Gender	/	/	/	/	/	−0.30^***^	−0.15^***^	0.19^***^	0.12^***^	−0.33^***^	
7	Age	19.15	1.23	/	/	/	0.05	0.07^**^	−0.12^***^	−0.15^***^	0.09^***^	−0.16^***^

Ske., skewness; Kur., kurtosis.

^*^p < 0.05, ^**^p < 0.01, ^***^p < 0.001.

### Hypothesis testing

**Figure 2:**
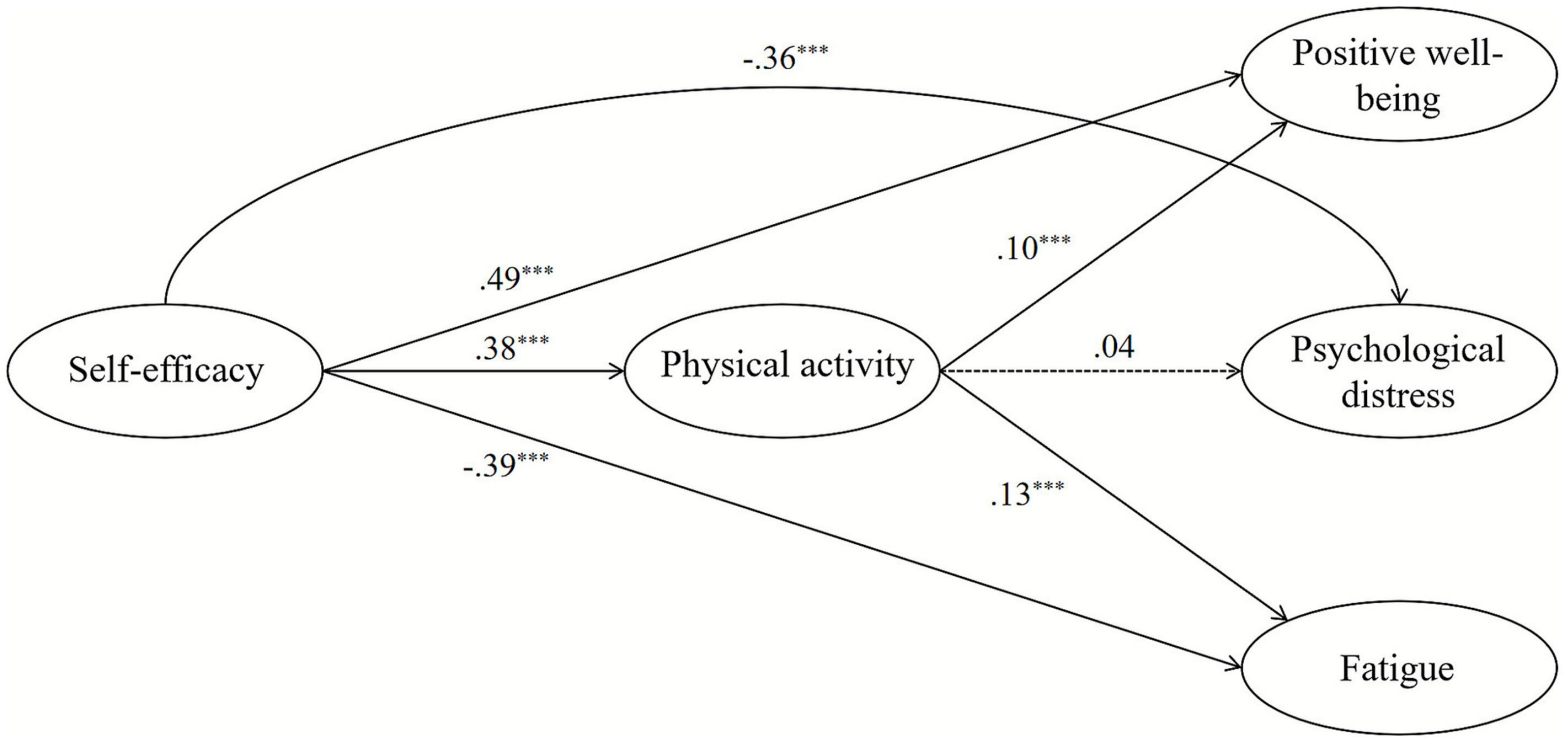
Final structural equation model. Solid line indicates path is significant. Dashed lines indicate path is not significant.

We tested our hypotheses using structural equation modeling (SEM). The model showed good fit with the data: *χ*^2^ (212) = 845.49, RMSEA = 0.04, CFI = 0.95, TLI = 0.94, and SRMR = 0.04. All fit indices met recommended standards and indicated the model adequately represented our data (Hu and Bentler, 1999). Results as illustrated in Table 2 and Figure 2, our findings support the hypothesized relationships.

**Table 2:** Results of structural equation modeling.

Path	*β*	SE	*p*
Self-efficacy → Physical activity	0.38	0.03	<0.01
Self-efficacy → Positive well-being	0.49	0.04	<0.01
Self-efficacy → Psychological distress	−0.36	0.04	<0.01
Self-efficacy → Fatigue	−0.39	0.04	<0.01
Physical activity → Positive well-being	0.10	0.03	<0.01
Physical activity → Psychological distress	0.04	0.03	0.22
Physical activity → Fatigue	0.13	0.03	<0.01
Gender → Self-efficacy	−0.32	0.03	<0.01
Gender → Physical activity	−0.21	0.03	<0.01
Gender → Positive well-being	0.03	0.03	0.28
Gender → Psychological distress	0.09	0.03	<0.01
Gender → Fatigue	0.02	0.03	0.56
Age → Self-efficacy	0.01	0.03	0.74
Age → Physical activity	0.03	0.02	0.22
Age → Positive well-being	0.04	0.03	0.12
Age → Psychological distress	−0.10	0.03	<0.01
Age → Fatigue	−0.14	0.03	<0.01

The SEM results supported most of our hypotheses concerning the mediating role of physical activity. As for H1, self-efficacy demonstrated a significant positive direct effect on physical activity (*β* = 0.38, *p* < 0.01). Furthermore, self-efficacy had significant direct effects on all subjective exercise experience outcomes. It positively influenced positive well-being (*β* = 0.49, *p* < 0.01) and negatively influenced both psychological distress (*β* = −0.36, *p* < 0.01) and fatigue (*β* = −0.39, *p* < 0.01). For H2, physical activity showed a significant positive direct effect on positive well-being (*β* = 0.10, *p* < 0.01) and a significant positive direct effect on fatigue (*β* = 0.13, *p* < 0.01). However, the path from physical activity to psychological distress was not significant (*β* = 0.04, *p* = 0.22). These findings indicate that physical activity serves as a partial mediator in the relationships between self-efficacy and positive well-being and between self-efficacy and fatigue, but not for psychological distress.

Regarding the control variables, gender exhibited significant associations with several key constructs. Being female was linked to lower levels of self-efficacy (*β* = −0.32, *p* < 0.01) and lower engagement in physical activity (*β* = −0.21, *p* < 0.01). Gender also had a significant direct positive effect on psychological distress (*β* = 0.09, *p* < 0.01), indicating that female students reported higher levels of distress. In contrast, age was not a significant predictor for self-efficacy, physical activity, or positive well-being. However, older age was associated with significantly lower levels of both psychological distress (*β* = −0.10, *p* < 0.01) and fatigue (*β* = −0.14, *p* < 0.01).

### The moderation role of gender

Prior to multi-group analysis, measurement invariance was assessed across gender for all latent constructs. As shown in Table 3, configural, metric, and scalar invariance models were tested. For self-efficacy, positive well-being, psychological distress, and fatigue, the changes in comparative fit index (ΔCFI) between nested models were all below the recommended threshold of |0.02|. Specifically, the ΔCFI values for self-efficacy, positive well-being, psychological distress, and fatigue were <−0.01, −0.01, <0.01, and <−0.01 for metric invariance, and −0.02, −0.02, 0.01, and <−0.01 for scalar invariance, respectively. This indicates that the factor structures, loadings, and intercepts were equivalent enough across genders to permit meaningful comparison of the structural paths, despite a significant *χ*^2^ difference for some constructs.

**Table 3:** Measurement invariance of the factors.

Variable	Model		*χ^2^*	df	RMSEA	CFI	SRMR	Model comparison	△RMAEA	△CFI
Self-efficacy	1	Configural invariance	227.64	40	0.09	0.95	0.04	/	/	/
	2	Metric invariance	241.70	47	0.08	0.93	0.05	1 and 2	−0.01	<−0.01
	3	Scalar invariance	312.92	54	0.09	0.91	0.06	2 and 3	0.01	−0.02
Positive well-being	1	Configural invariance	4.02	4	<0.01	1.00	0.01	/	/	/
	2	Metric invariance	14.20	7	0.04	0.99	0.05	1 and 2	0.04	−0.01
	3	Scalar invariance	37.97	10	0.06	0.97	0.04	2 and 3	0.02	−0.02
Psychological distress	1	Configural invariance	9.86	4	0.04	1.00	0.01	/	/	/
	2	Metric invariance	11.14	7	0.03	1.00	0.01	1 and 2	0.01	<0.01
	3	Scalar invariance	18.08	10	0.03	0.99	0.02	2 and 3	<0.01	0.01
Fatigue	1	Configural invariance	1.44	4	<0.01	1.00	<0.01	/	/	/
	2	Metric invariance	8.64	7	0.02	1.00	0.03	1 and 2	0.02	<−0.01
	3	Scalar invariance	13.99	10	0.02	1.00	0.03	2 and 3	0.01	<−0.01

**Figure 3:**
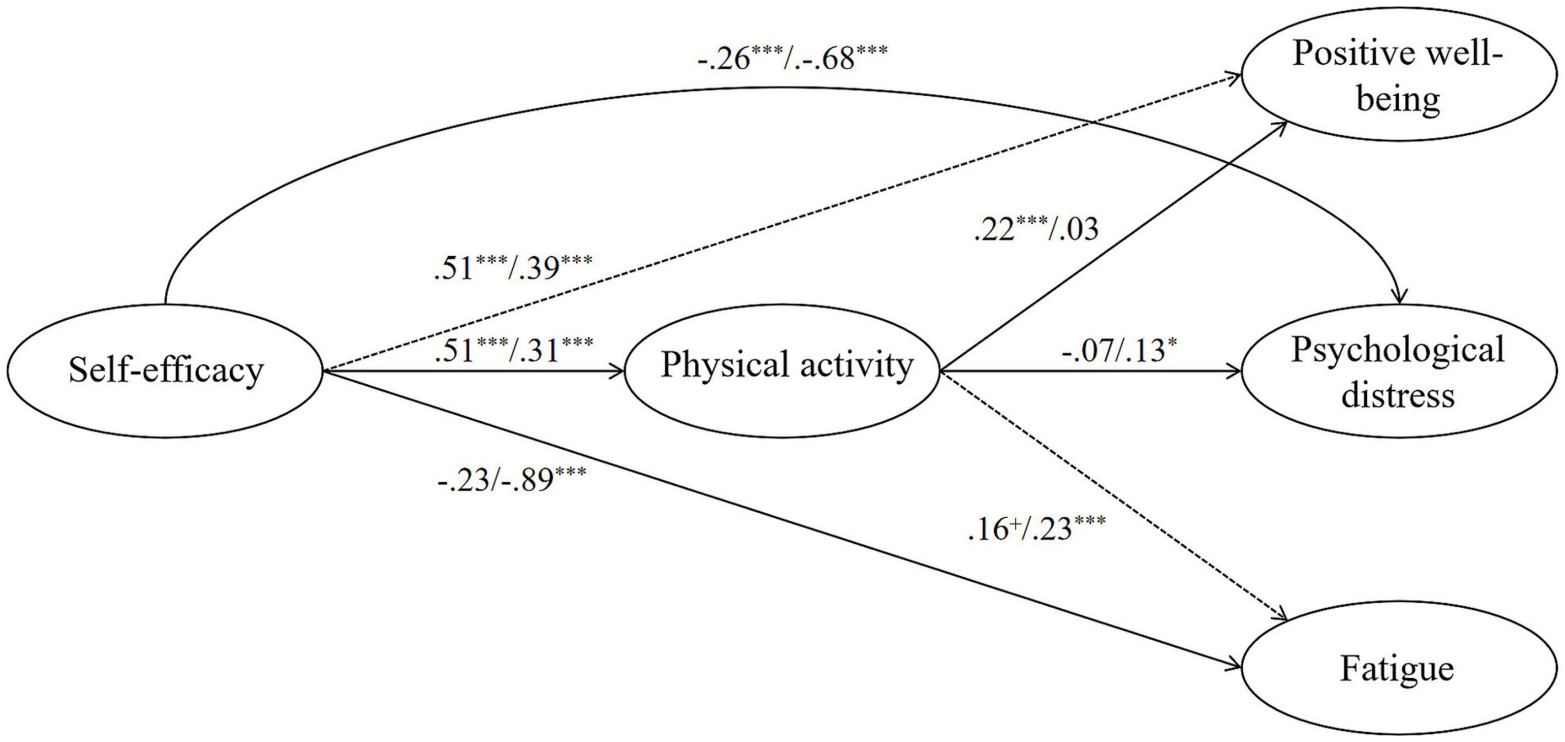
The results of moderation role of gender. Solid line indicates path is significant and dashed lines indicate path is not significant for gender difference.

The multi-group analysis revealed substantial gender differences in the structural model pathways (Table 4 and Figure 3). The effect of self-efficacy on physical activity was significantly stronger for male students (*β* = 0.51, *p* < 0.01) than for females (*β* = 0.31, *p* < 0.01), with a significant difference of 0.20 (*p* < 0.01). Furthermore, physical activity had a significant positive effect on positive well-being for males (*β* = 0.22, *p* < 0.01) but not for females (*β* = 0.03, *p* = 0.49), and this difference was statistically significant (*β* = 0.19, *p* = 0.02). Conversely, the direct negative effects of self-efficacy on psychological distress (*β* = −0.68, *p* < 0.01) and fatigue (*β* = −0.89, *p* < 0.01) were significantly stronger for females compared to males (*β* = −0.26, *p* = 0.04 and *β* = −0.23, *p* = 0.14, respectively). A notable gender-specific finding was that physical activity was positively associated with psychological distress for females (*β* = 0.13, *p* = 0.02), a relationship not present in males (*β* = −0.07, *p* = 0.34).

**Table 4:** The associations between factors and intention by gender.

Route	Male			Female			Comparison		
	*β*	SE	*p*	*β*	SE	*p*	*β*	SE	*p*
Self-efficacy → Physical activity	0.51	0.06	<0.01	0.31	0.03	<0.01	0.20	0.07	<0.01
Self-efficacy → Positive well-being	0.51	0.11	<0.01	0.59	0.06	<0.01	−0.08	0.11	0.50
Self-efficacy → Psychological distress	−0.26	0.13	0.04	−0.68	0.07	<0.01	0.42	0.14	<0.01
Self-efficacy → Fatigue	−0.23	0.15	0.14	−0.89	0.08	<0.01	0.67	0.17	<0.01
Physical activity → Positive well-being	0.22	0.07	<0.01	0.03	0.04	0.49	0.19	0.08	0.02
Physical activity → Psychological distress	−0.07	0.08	0.34	0.13	0.06	0.02	−0.21	0.10	0.04
Physical activity → Fatigue	0.16	0.10	0.10	0.23	0.07	<0.01	−0.07	0.12	0.59

## Discussion

This study examined the mediating role of physical activity between self-efficacy and subjective exercise experiences among Chinese college students, with a specific focus on gender differences. The key finding is that physical activity serves as a significant but partial mediator, and its role is highly gender-specific. For males, the primary pathway to enhanced well-being is through self-efficacy boosting physical activity. For females, self-efficacy exerts a more direct and potent effect in reducing psychological distress and fatigue, while their relationship with physical activity appears more complex. These results underscore the necessity for gender-tailored strategies to improve student exercise experiences, moving beyond a one-size-fits-all approach.

### Theoretical interpretation and integration

Our findings offer a nuanced perspective on social cognitive theory (SCT) by delineating the specific pathways through which self-efficacy influences subjective exercise experiences. The significant positive effect of self-efficacy on physical activity is a cornerstone finding that aligns robustly with SCT and a substantial body of prior research (Bandura, 1997; Olander et al., 2013). This confirms that students’ confidence in their ability to exercise is a powerful driver of their actual behavioral engagement, a relationship consistently observed across different populations.

Furthermore, the strong direct effects of self-efficacy on all three subjective exercise outcomes reinforce the centrality of cognitive processes in SCT. The direct boost to positive well-being and the direct reductions in psychological distress and fatigue suggest that self-efficacy does not only operate through behavior. It also provides a potent psychological resource that directly enhances how students feel during exercise, likely by shaping their appraisal of physical sensations and challenges (McAuley and Blissmer, 2000). This pattern of direct effects is consistent with studies linking self-efficacy to improved affective states independent of activity level (Buecker et al., 2021).

A key novel contribution of our study is the precise mapping of physical activity as a partial mediator. We found that physical activity transmits the benefits of self-efficacy to positive well-being and, unexpectedly, is linked to higher fatigue. The mediation for positive well-being is intuitive and fits within the SCT framework where behavior leads to reinforcing outcomes (McAuley and Blissmer, 2000). However, the mediating role for fatigue introduces a more complex narrative, suggesting that the behavior motivated by high self-efficacy also carries a physical cost, which is an under-discussed nuance in the SCT model as applied to exercise (Basso and Suzuki, 2016).

Perhaps our most significant theoretical advancement is the clear boundary condition we identified. The lack of a significant mediating role of physical activity between self-efficacy and psychological distress is a critical finding. It indicates that the mechanism through which self-efficacy alleviates exercise-related distress is primarily direct and cognitive-emotional, rather than behavioral. This suggests that other unmeasured factors, such as coping strategies or social support, may be more salient in this particular pathway (Liu et al., 2023), inviting a theoretical extension of SCT to include these elements for a more complete understanding of distress reduction (Liu et al., 2023).

### Distinct psychological pathways by gender

Our multi-group analysis reveals that the psychological architecture linking self-efficacy, behavior, and subjective experience is fundamentally different for male and female students. These findings move beyond simply documenting mean-level differences and illuminate the distinct operative mechanisms within each group.

For male students, the pathway is predominantly behavioral and agentic. The significantly stronger effect of self-efficacy on physical activity for males compared to females aligns with literature suggesting that male exercise participation is often more directly driven by confidence in physical abilities and a focus on mastery (Guthold et al., 2018). Crucially, physical activity subsequently serves as a reliable vehicle for enhancing positive well-being in males (Brewer, 2018). This chain of self-efficacy driving behavior which in turn generates positive affect represents a clear and efficient pathway that is strongly consistent with core SCT principles.

For female students, the model is more complex and psychologically nuanced that the most striking finding is the profound direct protective effect of self-efficacy. The strong negative paths from self-efficacy to psychological distress and fatigue suggest that for female students, self-belief functions as a critical internal buffer against negative exercise experiences. This indicates that interventions for females might yield greater returns by focusing on building this internal resource rather than solely emphasizing behavioral output. This heightened direct effect resonates with studies indicating that women’s physical activity experiences are more heavily influenced by psychological and social factors, such as social physique anxiety and internalization of cultural body ideals (Sabiston et al., 2019). Furthermore, the dominant role of self-efficacy in directly mitigating negative states is consistent with research highlighting that cognitive-appraisal processes are central to understanding women’s affective responses to exercise, often superseding the role of mere behavioral participation (Ekkekakis et al., 2018).

A novel and critical discovery is the unique positive association between physical activity and psychological distress found exclusively in the female cohort. This counterintuitive relationship suggests that for some female students, the act of being physically active may be associated with increased feelings of distress. This finding challenges the universal assumption that physical activity is inherently psychologically beneficial. It may be explained by factors such as social physique anxiety, where exercise settings trigger body image concerns (Sabiston et al., 2019), or experiences of perceived pressure and judgment (Liu et al., 2023).

Thus, our results demonstrate that gender acts as a powerful moderator of the entire psychological process. Males largely follow a behavioral pathway where confidence enables action that leads to reward. Females, conversely, exhibit a potent direct pathway where self-efficacy powerfully mitigates negative states, while their relationship with the behavior itself can be psychologically complex and potentially fraught. This delineation of divergent pathways provides a sophisticated, evidence-based foundation for developing genuinely tailored interventions.

### Practical implications for targeted interventions

Our findings provide a clear, evidence-based roadmap for developing gender-specific physical activity interventions for college students. A universal approach is inadequate given the fundamentally different psychological pathways we identified.

For male students, interventions should leverage the strong behavioral pathway we observed. Programmers should focus on building self-efficacy through mastery experiences, such as progressive skill-building in sports or fitness training, to directly increase physical activity participation. This increased activity will then naturally serve as a primary source of positive well-being for them. This approach aligns with established methods for promoting male physical activity (Olander et al., 2013).

For female students, the intervention strategy must be different. The core focus should be on developing self-efficacy in a supportive context that minimizes social evaluation. Instead of prioritizing sheer activity volume, programs should foster confidence through activities like yoga, dance, or non-competitive group fitness that emphasize personal accomplishment and body awareness. This directly targets the powerful protective effect self-efficacy has against distress and fatigue, which is the most significant lever for improving their subjective experience (Liu et al., 2023).

Critically, our discovery that physical activity can correlate with distress in females necessitates an environmental shift. Practitioners must actively create safe, non-judgmental spaces for physical activity that address potential sources of anxiety like body image concerns. This ensures that the act of being active does not inadvertently undermine the psychological benefits gained from enhanced self-efficacy.

### Limitations and future research

This study has several limitations that should be considered. First, the cross-sectional nature of our data prevents definitive conclusions about causality. While our model is grounded in theory, longitudinal or experimental designs are necessary to confirm the proposed directional relationships between self-efficacy, physical activity, and subjective experiences over time.

Secondly, the generalizability of our findings may be constrained by the use of a convenience sample from a single university and the notable gender imbalance. Future studies should employ stratified sampling across multiple institutions to obtain more balanced gender representation and enhance the external validity of the results.

Finally, the unexpected positive association between physical activity and psychological distress in female students highlights a critical area for further inquiry. Future research should use qualitative methods or incorporate specific moderating variables, such as social physique anxiety or motivational climate, to better understand the contextual factors that transform physical activity into a source of distress for some women.

## Conclusion

This study moves beyond establishing simple relationships to delineate the nuanced pathways through which self-efficacy influences how college students feel during exercise. By confirming the gendered nature of these psychological processes, we provide a robust evidence base for developing more effective, tailored interventions to promote not only physical activity but also positive and sustainable subjective exercise experiences for all students.

## Data Availability

The datasets presented in this study can be found in online repositories. The names of the repository/repositories and accession number(s) can be found in the article/supplementary material.

## References

[ref1] Ashford S. Edmunds J. French D. P. (2010). What is the best way to change self-efficacy to promote lifestyle and recreational physical activity? A systematic review with meta-analysis. Br. J. Health Psychol. 15, 265–288. doi: 10.1348/135910709x461752, 19586583

[ref2] Bandura A. (1997). Self-efficacy: the exercise of control. New York: W.H. Freeman.

[ref3] Bandura A. (2004). Health promotion by social cognitive means. Health Educ. Behav. 31, 143–164. doi: 10.1177/1090198104263660, 15090118

[ref4] Bandura A. Wessels S. (1994). “Self-efficacy” in Encyclopedia of human behavior (New York: Academic Press).

[ref5] Basso J. C. Suzuki W. A. (2016). The effects of acute exercise on mood, cognition, neurophysiology, and neurochemical pathways: a review. Brain Plast. 2, 127–152. doi: 10.3233/bpl-160040 PMC5928534 29765853

[ref6] Biedenweg K. Meischke H. Bohl A. Hammerback K. Williams B. Poe P. . (2014). Understanding older adults’ motivators and barriers to participating in organized programs supporting exercise behaviors. J. Prim. Prev. 35, 1–11. doi: 10.1007/s10935-013-0331-2, 24214654

[ref7] Brewer H. J. (2018). “Foundations of physical activity and health promotion in early childhood” in Physical activity and health promotion in the early years: effective strategies for early childhood educators (Cham: Springer), 3–17.

[ref8] Buecker S. Simacek T. Ingwersen B. Terwiel S. Simonsmeier B. A. (2021). Physical activity and subjective well-being in healthy individuals: a meta-analytic review. Health Psychol. Rev. 15, 574–592. doi: 10.1080/17437199.2020.1760728 32452716

[ref9] Chaput J.-P. Willumsen J. Bull F. Chou R. Ekelund U. Firth J. . (2020). 2020 WHO guidelines on physical activity and sedentary behaviour for children and adolescents aged 5–17 years: summary of the evidence. Int. J. Behav. Nutr. Phys. Act. 17:141. doi: 10.1186/s12966-020-01037-z, 33239009 PMC7691077

[ref10] Chen H. Dai J. Gao Y. (2019). Measurement invariance and latent mean differences of the Chinese version physical activity self-efficacy scale across gender and education levels. J. Sport Health Sci. 8, 46–54. doi: 10.1016/j.jshs.2017.01.004, 30719383 PMC6349578

[ref11] Craig C. L. Marshall A. L. Sjöström M. Bauman A. E. Booth M. L. Ainsworth B. E. . (2003). International physical activity questionnaire: 12-country reliability and validity. Med. Sci. Sports Exerc. 35, 1381–1395, 12900694 10.1249/01.MSS.0000078924.61453.FB

[ref12] Dishman R. K. Berthoud H. Booth F. W. Cotman C. W. Edgerton V. R. Fleshner M. R. . (2006). Neurobiology of exercise. Obesity 14, 345–356. doi: 10.1038/oby.2006.46 16648603

[ref13] Eather N. Wade L. Pankowiak A. Eime R. (2023). The impact of sports participation on mental health and social outcomes in adults: a systematic review and the ‘mental health through sport’ conceptual model. Syst. Rev. 12:102. doi: 10.1186/s13643-023-02264-8, 37344901 PMC10286465

[ref14] Ekkekakis P. Zenko Z. Ladwig M. A. Hartman M. E. (2018). “Affect as a potential determinant of physical activity and exercise” in Affective determinants of health behavior (New York: Oxford Academic), 237–261.

[ref15] Esmaeilzadeh S. Rodriquez-Negro J. Pesola A. J. (2022). A greater intrinsic, but not external, motivation toward physical activity is associated with a lower sitting time. Front. Psychol. 13:888758. doi: 10.3389/fpsyg.2022.888758, 35645933 PMC9133934

[ref16] Gauvin L. Spence J. (1998). “Measurement of exercise-induced changes in feeling states, affect, mood, and emotions” in Advances in sport and exercise psychology measurement (Morgantown, WV: Fitness Information Technology, Inc.), 325–336.

[ref17] Guthold R. Stevens G. A. Riley L. M. Bull F. C. (2018). Worldwide trends in insufficient physical activity from 2001 to 2016: a pooled analysis of 358 population-based surveys with 1·9 million participants. Lancet Glob. Health 6, e1077–e1086. doi: 10.1016/S2214-109X(18)30357-7, 30193830

[ref18] He L. Li Y. Chen Z. (2022). The effect of subjective exercise experience on exercise behavior and amount of exercise in children and adolescents: the mediating effect of exercise commitment. Int. J. Environ. Res. Public Health 19:10829. doi: 10.3390/ijerph191710829, 36078545 PMC9518043

[ref19] Hong J.-T. Chen S.-T. Tang Y. Cao Z.-B. Zhuang J. Zhu Z. . (2020). Associations between various kinds of parental support and physical activity among children and adolescents in shanghai, China: gender and age differences. BMC Public Health 20:1161. doi: 10.1186/s12889-020-09254-8, 32711483 PMC7382138

[ref20] Hu L. Bentler P. M. (1999). Cutoff criteria for fit indexes in covariance structure analysis: conventional criteria versus new alternatives. Struct. Equ. Model. Multidiscip. J. 6, 1–55. doi: 10.1080/10705519909540118

[ref21] Jago R. Salway R. House D. Beets M. Lubans D. R. Woods C. . (2023). Rethinking children’s physical activity interventions at school: a new context-specific approach. Front. Public Health 11:1149883. doi: 10.3389/fpubh.2023.1149883, 37124783 PMC10133698

[ref22] Kandola A. Ashdown-Franks G. Hendrikse J. Sabiston C. M. Stubbs B. (2019). Physical activity and depression: towards understanding the antidepressant mechanisms of physical activity. Neurosci. Biobehav. Rev. 107, 525–539. doi: 10.1016/j.neubiorev.2019.09.040, 31586447

[ref23] Li Y.-C. Joshi D. King-Dowling S. Hay J. Faught B. E. Cairney J. (2018). The longitudinal relationship between generalized self-efficacy and physical activity in school-aged children. Eur. J. Sport Sci. 18, 569–578. doi: 10.1080/17461391.2018.1430852 29400618

[ref24] Liu Y. Ge X. Li H. Zhang E. Hu F. Cai Y. . (2023). Physical activity maintenance and increase in Chinese children and adolescents: the role of intrinsic motivation and parental support. Front. Public Health 11:1175439. doi: 10.3389/fpubh.2023.1175439, 37583889 PMC10424444

[ref25] McAuley E. Blissmer B. (2000). Self-efficacy determinants and consequences of physical activity. Exerc. Sport Sci. Rev. 28, 85–88. 10902091

[ref26] MeAuley E. Courneya K. S. (1994). The subjective exercise experiences scale (SEES): development and preliminary validation. J. Sport Exerc. Psychol. 16, 163–177. doi: 10.1123/jsep.16.2.163

[ref27] Olander E. K. Fletcher H. Williams S. Atkinson L. Turner A. French D. P. (2013). What are the most effective techniques in changing obese individuals’ physical activity self-efficacy and behaviour: a systematic review and meta-analysis. Int. J. Behav. Nutr. Phys. Act. 10:29. doi: 10.1186/1479-5868-10-29, 23452345 PMC3639155

[ref28] Osborne J. W. (2012). Best practices in data cleaning: a complete guide to everything you need to do before and after collecting your data. Thousand Oaks, CA: SAGE publications.

[ref29] Podsakoff P. M. MacKenzie S. B. Lee J.-Y. Podsakoff N. P. (2003). Common method biases in behavioral research: a critical review of the literature and recommended remedies. J. Appl. Psychol. 88:879. doi: 10.1037/0021-9010.88.5.879, 14516251

[ref30] Reed J. Ones D. S. (2006). The effect of acute aerobic exercise on positive activated affect: a meta-analysis. Psychol. Sport Exerc. 7, 477–514. doi: 10.1016/j.psychsport.2005.11.003

[ref31] Rovniak L. S. Anderson E. S. Winett R. A. Stephens R. S. (2002). Social cognitive determinants of physical activity in young adults: a prospective structural equation analysis. Ann. Behav. Med. 24, 149–156. doi: 10.1207/S15324796ABM2402_12, 12054320

[ref32] Ryan R. M. Deci E. L. (2000). Self-determination theory and the facilitation of intrinsic motivation, social development, and well-being. Am. Psychol. 55:68. doi: 10.1037/0003-066x.55.1.68, 11392867

[ref33] Sabiston C. Pila E. Vani M. Thogersen-Ntoumani C. (2019). Body image, physical activity, and sport: a scoping review. Psychol. Sport Exerc. 42, 48–57. doi: 10.1016/j.psychsport.2018.12.010

[ref34] van Sluijs E. M. Ekelund U. Crochemore-Silva I. Guthold R. Ha A. Lubans D. . (2021). Physical activity behaviours in adolescence: current evidence and opportunities for intervention. Lancet 398, 429–442. doi: 10.1016/S0140-6736(21)01259-9 34302767 PMC7612669

[ref35] Warburton D. E. Bredin S. S. (2017). Health benefits of physical activity: a systematic review of current systematic reviews. Curr. Opin. Cardiol. 32, 541–556. doi: 10.1097/HCO.0000000000000437, 28708630

[ref36] Warner L. M. Schüz B. Wolff J. K. Parschau L. Wurm S. Schwarzer R. (2014). Sources of self-efficacy for physical activity. Health Psychol. 33, 1298–1308. doi: 10.1037/hea0000085, 24707842

[ref37] White R. L. Babic M. J. Parker P. D. Lubans D. R. Astell-Burt T. Lonsdale C. (2017). Domain-specific physical activity and mental health: a meta-analysis. Am. J. Prev. Med. 52, 653–666. doi: 10.1016/j.amepre.2016.12.008, 28153647

[ref38] Williams D. M. Rhodes R. Conner M. (2018). “Psychological hedonism, hedonic motivation, and health behavior” in Affective determinants of health behavior (New York: Oxford Academic), 205–234.

[ref39] Xiong B. Skitmore M. Xia B. (2015). A critical review of structural equation modeling applications in construction research. Autom. Constr. 49, 59–70. doi: 10.1016/j.autcon.2014.09.006

[ref40] Yan W. Wang Y. Yuan Y. Farid M. Zhang P. Peng K. (2024). Timing matters: a longitudinal study examining the effects of physical activity intensity and timing on adolescents’ mental health outcomes. J. Youth Adolesc. 53, 2320–2331. doi: 10.1007/s10964-024-02011-9, 38767791

[ref41] Zhu Z. Yang Y. Kong Z. Zhang Y. Zhuang J. (2017). Prevalence of physical fitness in Chinese school-aged children: findings from the 2016 physical activity and fitness in China—the youth study. J. Sport Health Sci. 6, 395–403. doi: 10.1016/j.jshs.2017.09.003, 30356643 PMC6189247

